# Resistant hypertension and epileptic seizures: a possible modulatory role of renal denervation—a case report

**DOI:** 10.1093/ehjcr/ytag592

**Published:** 2026-08-11

**Authors:** Antonio Bustos-Merlo, Alberto Fajardo-Muñoz, Teresa Bretones-Del Pino, Eduardo Molina-Navarro, Fernando Jaén-Águila

**Affiliations:** Department of Internal Medicine, Hospital Virgen de las Nieves, Avenida de las Fuerzas Armadas, 18014 Granada, Spain; Instituto de Investigación Biosanitaria ibs.GRANADA, 18016 Granada, Spain; Department of Internal Medicine, Hospital Virgen de las Nieves, Avenida de las Fuerzas Armadas, 18014 Granada, Spain; Department of Cardiology, Hospital Virgen de las Nieves, Avenida de las Fuerzas Armadas, 18014 Granada, Spain; Department of Cardiology, Hospital Virgen de las Nieves, Avenida de las Fuerzas Armadas, 18014 Granada, Spain; Department of Internal Medicine, Hospital Virgen de las Nieves, Avenida de las Fuerzas Armadas, 18014 Granada, Spain; Instituto de Investigación Biosanitaria ibs.GRANADA, 18016 Granada, Spain

**Keywords:** Resistant hypertension, Autonomic epilepsy, Renal denervation, Sympathetic modulation, Drug-resistant seizures, Case report

## Abstract

**Background:**

Renal denervation (RDN) is an emerging therapy for selected patients with resistant hypertension. Its broader autonomic effects may extend beyond blood pressure regulation.

**Case summary:**

A 57-year-old man with resistant hypertension treated with six antihypertensive drugs and recurrent autonomic and generalized seizures underwent radiofrequency RDN. At 3 months, ambulatory blood pressure monitoring showed sustained improvement (24 h mean 139/87 mmHg), enabling therapy de-escalation. Generalized tonic–clonic seizures resolved, and autonomic/focal events markedly decreased without changes in antiepileptic medication.

**Discussion:**

This case illustrates a potential extrapressor effect of RDN mediated by modulation of sympathetic overactivity. Although causality cannot be drawn from a single case, the parallel neurological improvement supports exploring RDN’s role in conditions involving autonomic dysregulation, including certain epileptic syndromes.

Learning points

- Modulation of sympathetic hyperactivity via renal denervation (RDN) may influence seizure activity in patients with autonomic epilepsy.
- In selected cases, RDN may provide neurological benefits beyond blood pressure reduction, suggesting a potential role in disorders involving autonomic dysregulation.

## Introduction

Renal denervation (RDN) is a minimally invasive intervention that disrupts renal sympathetic nerve fibres and is recommended for selected patients with resistant hypertension (Class IIb) by current European guidelines.^1^ Resistant hypertension is defined as blood pressure (BP) remaining above target despite treatment with ≥3 antihypertensive drugs of different classes, including a diuretic, or requiring ≥4 drugs to achieve control.

Beyond its antihypertensive effects, RDN reduces global sympathetic outflow, potentially influencing conditions in which autonomic dysfunction plays a pathophysiological role.

Autonomic manifestations are increasingly recognized within the epilepsy spectrum, particularly in temporal and insular lobe epilepsy, where altered central autonomic regulation may modulate cortical excitability.^2^

We report a case of resistant hypertension and drug-resistant epilepsy with prominent autonomic features, showing parallel clinical improvement following RDN, raising the hypothesis of a shared mechanism linked to sympathetic dysregulation.

## Summary figure

**Figure:**
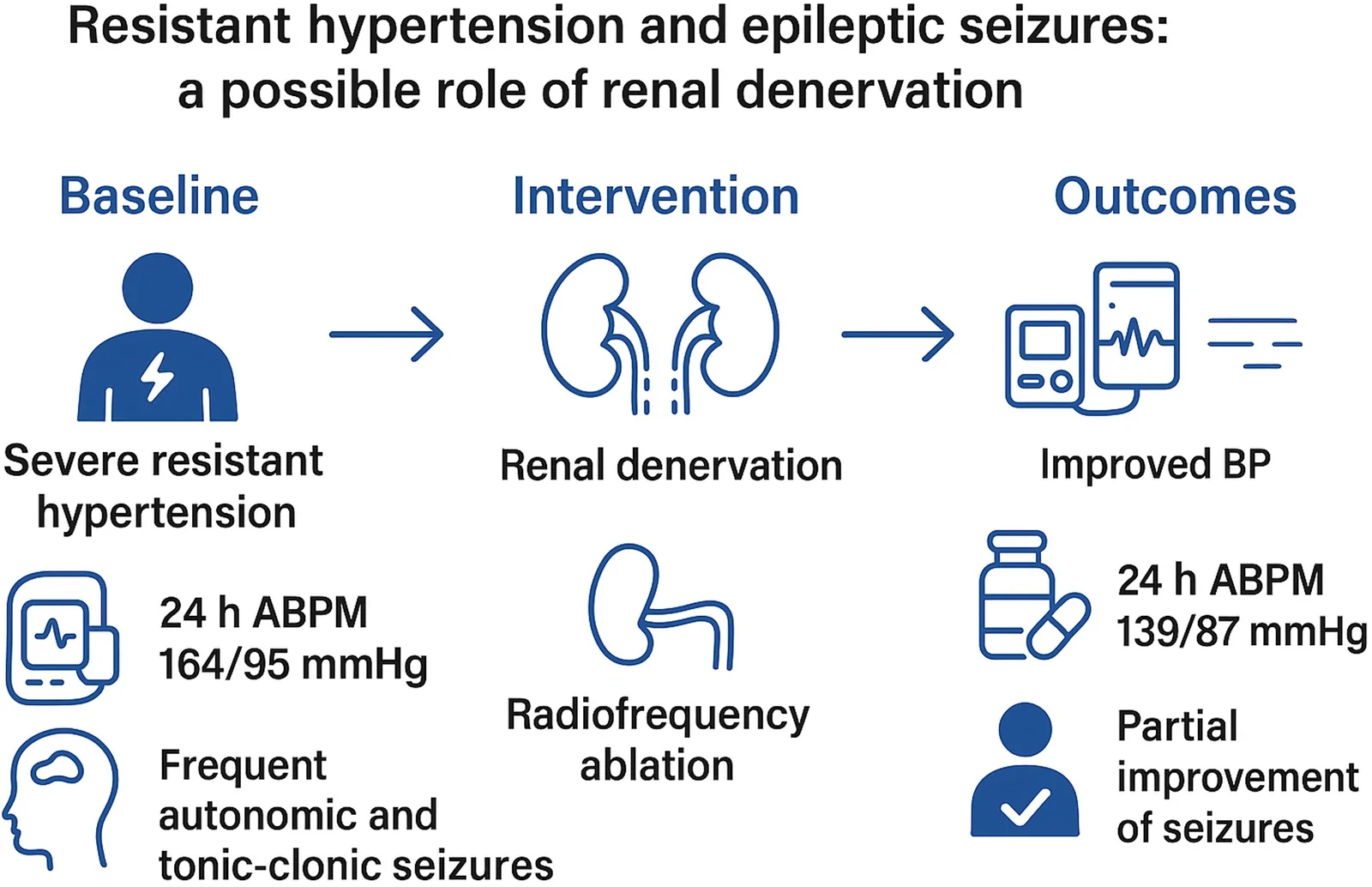


## Case presentation

A 57-year-old man with long-standing hypertension was referred for evaluation of persistently elevated BP despite treatment with six antihypertensive drugs (olmesartan 40 mg/day, amlodipine 10 mg/day, hydrochlorothiazide 25 mg/day, spironolactone 100 mg/day, doxazosin 16 mg every 12 h, and nebivolol 10 mg/day).

Medication adherence was confirmed, secondary causes of hypertension were excluded, and toxicology screening was negative. Physical examination was unremarkable except for obesity (body mass index 33 kg/m^2^) and elevated BP.

Ambulatory blood pressure monitoring (ABPM) showed sustained severe hypertension with a nondipping pattern:

- 24 h mean: 164/95 mmHg,
- Daytime mean: 169/98 mmHg, and
- Night-time mean: 151/89 mmHg.

Home measurements frequently exceeded 220/120 mmHg, particularly under stress.

Comorbidities included obesity, obstructive sleep apnoea under stable continuous positive airway pressure (CPAP) therapy, and an adjustment disorder.

The patient reported daily episodes characterized by autonomic symptoms including diaphoresis, palpitations, and epigastric discomfort, followed by transient impairment of awareness and occasional motor automatisms. Some episodes progressed to bilateral tonic–clonic seizures, occasionally associated with sphincter release.

Brain magnetic resonance imaging and cerebrospinal fluid analysis were unremarkable. Routine electroencephalography (EEG) demonstrated interictal epileptiform discharges predominantly in the left temporal region.

Seizures were classified according to International League Against Epilepsy criteria as focal seizures with autonomic features, with occasional secondary generalization. The stereotyped semiology and EEG findings supported an epileptic origin, making alternative diagnoses such as psychogenic nonepileptic events unlikely.

**Figure 1:**
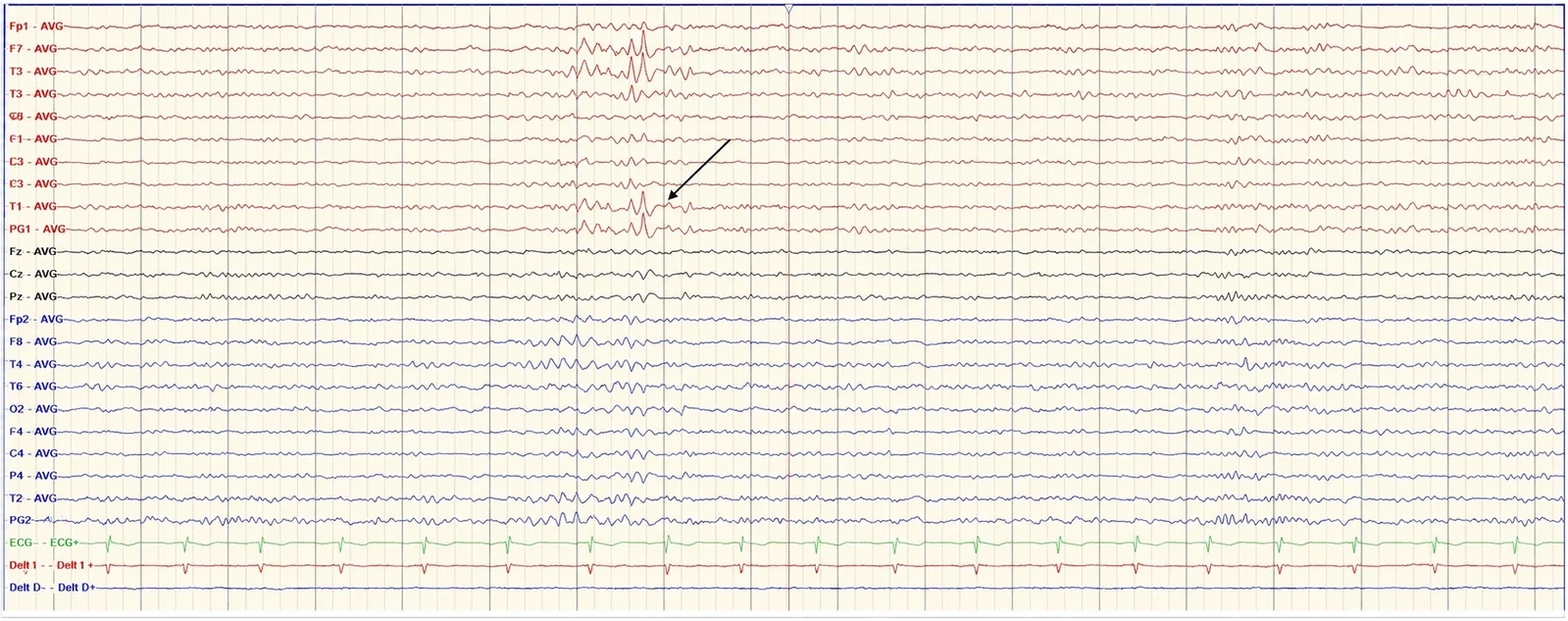
Interictal electroencephalography showing left temporal epileptiform discharges (arrow), characterized by sharp waves and spike-and-wave complexes consistent with focal cortical irritability.

The patient had previously received multiple antiepileptic drugs, including lamotrigine, valproate, and brivaracetam, at maximally tolerated doses and appropriate durations. Serum levels, when assessed, were within therapeutic ranges. Despite this, seizure control remained suboptimal, fulfilling criteria for drug-resistant epilepsy (*Figure 1*).

Given the diagnosis of resistant hypertension and suspected sympathetic overactivity contributing to both cardiovascular and neurological manifestations, radiofrequency RDN was performed using the Symplicity Spyral™ catheter system. Thirty-one ablations were delivered to the left renal artery and 34 to the right. The procedure was uneventful.

## Follow-up

At 3-month follow-up, BP control improved significantly, with no home measurements exceeding 160/90 mmHg. Antihypertensive therapy was reduced from six to four agents.

ABPM showed partial normalization:

- 24 h: 139/87 mmHg,
- Daytime: 138/87 mmHg, and
- Night-time: 141/87 mmHg (nondipping pattern persisted).

Neurologically, generalized tonic–clonic seizures resolved completely, and autonomic/focal events markedly decreased without any modification of antiepileptic therapy.

The patient reported substantial improvement in daily functioning and quality of life (*Table 1*).

**Table 1:** Monthly evolution of blood pressure and seizure activity before and after renal denervation (RDN)

	−6 months	−3 months RDN (0)	+3 months	+6 months
Mean SBP (mmHg)	200	200	153	147
Mean DBP (mmHg)	145	133	95	93
Tonic–clonic seizures (no sphincter release)	43	53	49	47
Tonic–clonic seizures (with sphincter release)	2	2	0	0
Absence seizures	77	82	68	64
Partial rigidity episodes	65	69	44	35
Days with functional limitation	8	11	5	6

All data were extracted from the patient's clinical diary and verified during scheduled follow-up visits. DBP, diastolic blood pressure; SBP, systolic blood pressure.

## Discussion

Resistant hypertension is strongly associated with sympathetic hyperactivity. RDN reduces both renal and central sympathetic outflow, potentially exerting effects beyond BP lowering.^3–5^

In this case, the patient presented with focal epilepsy with prominent autonomic features and EEG findings suggestive of temporal lobe involvement. The insular cortex and related limbic structures play a key role in integrating autonomic and cortical activity, and have been implicated in both seizure generation and cardiovascular regulation.

Experimental and clinical evidence suggests that increased sympathetic activity may enhance cortical excitability and contribute to seizure susceptibility.^6^ Renal afferent denervation may modulate central autonomic networks through reduced signalling to brainstem nuclei such as the nucleus tractus solitarius and locus coeruleus, with downstream projections to higher cortical regions including the insular cortex.^7^ This provides a plausible neurobiological framework linking renal sympathetic modulation with changes in cortical excitability.

Notably, the reduction in seizure frequency occurred without changes in antiepileptic therapy, suggesting a possible independent or complementary mechanism. However, this observation must be interpreted with caution.

The temporal association between RDN and neurological improvement does not establish causality. Alternative explanations should be considered, including regression to the mean, placebo or expectancy effects, natural variability in seizure frequency, and indirect effects related to improved BP control or reduced psychological stress. Additionally, factors such as stable CPAP adherence may have contributed to clinical improvement and cannot be fully disentangled.

Current European guidelines recognize RDN as a treatment option for selected patients with resistant hypertension, although its effects beyond BP reduction remain incompletely characterized. Trials such as SPYRAL HTN-OFF MED have focused on haemodynamic outcomes and did not assess neurological endpoints.^8^

This case highlights a potential interaction between autonomic regulation and epileptic activity and supports further investigation into the broader systemic effects of RDN. Prospective studies are needed to clarify whether modulation of sympathetic activity may have therapeutic implications in selected neurological conditions.

## Patient’s perspective

The patient reported a marked improvement in quality of life and daily functioning after the intervention, particularly due to the cessation of generalized seizures and the reduction of daily autonomic episodes.

## Data Availability

The data underlying this article are available within the article. No additional data are available.

## Lead author biography

Antonio Bustos-Merlo is a specialist in Internal Medicine at Hospital Universitario Virgen de las Nieves, Granada, Spain. He completed his medical training at the University of Granada. His clinical and research interests focus on hypertension, cardiovascular risk, and heart failure. He has contributed to multiple case reports and studies on innovative interventions for resistant hypertension and autonomic dysfunction. His work integrates clinical practice with research on autonomic modulation and its cardiovascular and neurological implications, aiming to improve the management of patients with complex cardiovascular conditions.

## Notes

**Consent:** Written informed consent was obtained from the patient for publication of this case report and all accompanying images. The authors confirm that written consent for submission and publication of this case report, including images and associated text, has been obtained from the patient, in line with COPE guidance.
